# C3 glomerulonephritis associated with monoclonal gammopathy of renal significance: a diagnostic and therapeutic challenge

**DOI:** 10.1590/2175-8239-JBN-2024-0106en

**Published:** 2025-02-07

**Authors:** Bárbara Beirão, Mariana Freitas, Natália Silva, Patrícia Ferraz, Catarina Prata, Teresa Morgado

**Affiliations:** 1 Centro Hospitalar de Trás-os-Montes e Alto Douro, Hospital de Vila Real, Serviço de Nefrologia, Vila Real, Portugal.; 2 Centro Hospitalar de Trás-os-Montes e Alto Douro, Hospital de Vila Real, Serviço de Hematologia, Vila Real, Portugal.

**Keywords:** Monoclonal Gammopathy, C3 Glomerulopathy, Monoclonal Gammopathy of Renal Significance, Targeted Therapy

## Abstract

C3 glomerulopathy represents a heterogeneous group of glomerulopathies characterized by hyperactivity of the alternative complement pathway. Although the pathophysiology is incompletely understood, an association between this disease and monoclonal gammopathies is increasingly recognized, especially in older individuals. There is still some uncertainty regarding the best treatment for patients with monoclonal gammopathy-associated C3 glomerulopathy. However, recent evidence suggests that myeloma-targeted therapies are associated with improved renal outcomes compared with conservative or conventional immunosuppressive therapies. This case report describes the clinical course and long-term follow-up of a patient with monoclonal gammopathy-associated C3 glomerulonephritis treated with myeloma-targeted therapy.

## Introduction

The term monoclonal gammopathy of renal significance (MGRS) describes hematological conditions where the production and secretion of a monoclonal immunoglobulin (mIg) causes kidney damage^1^. These hematological disorders do not meet diagnostic criteria for multiple myeloma or lymphoproliferative disease and, thus, do not meet previously defined hematological criteria for targeted treatment^1^.

The spectrum of MGRS-associated kidney diseases is broad. Kidney injury can be caused directly by the deposition of the monoclonal protein or, less commonly, by dysregulation of the alternative complement pathway (AP)^1–3^. In this indirect mechanism, monoclonal Ig acts as an autoantibody, activating AP by inhibiting complement regulatory proteins, and there are no Ig deposits in the kidney^1,3^. The best example of an MGRS-associated disorder with absent or sparse monoclonal-Ig deposition is C3 glomerulopathy (C3G), including dense deposit disease (DDD) and C3 glomerulonephritis (C3GN)^1^.

Although C3G is rare, several studies have shown a high prevalence of monoclonal gammopathy in patients older than 50 years with C3G (reaching 65% in some series)^4^, much higher than the prevalence of monoclonal gammopathy in the general population (4.2%)^5,6^.

C3G is a histopathological diagnosis, characterized by C3 dominant deposition on immunofluorescent staining. Presentation is variable, ranging from asymptomatic hematuria to rapidly progressive glomerulonephritis^7^.

Currently, there is still some uncertainty regarding the best treatment for patients with mIg-C3GN. However, evidence from a few case series and case reports suggests that myeloma-targeted therapies are associated with better renal outcomes compared to conservative or conventional immunosuppressive therapies^4,8,9^.

This case report describes the clinical course and outcomes of a patient with C3GN-associated MGRS treated with clone-directed therapy.

## Case Report

A 61-year-old male presented to our service with history of heart failure (LVEF 46%) after two hospitalizations for myopericarditis with cardiogenic shock in 2018 and 2021. Myocardial biopsy performed in 2018 showed a discrete, non-specific lymphocytic infiltrate. There was no evidence of immune or neoplastic etiology, and a diagnosis of viral myopericarditis was assumed. He had an IgG/ kappa monoclonal gammopathy of undetermined significance (MGUS) diagnosed in 2021 (M-spike of 0.38 g/dL on serum protein electrophoresis/ immunofixation and free light chain ratio kappa/ lambda of 5.47).

In January 2022, he presented with erythrocyturia of 25–50/hpf, proteinuria of 2.5g/ day (albuminuria 1700 mg/day), and preserved renal function (CrS 1.1/ Ur 65 mg/dL, creatinine clearance 94 mL/min). The diagnosis of IgG/ kappa monoclonal gammopathy was established based on serum and urine immunofixation results: M protein of 0.4 g/dL, serum kappa light chain level of 11.8 mg/L, and serum lambda light chain level of 1.16 mg/L, with a corresponding ratio of 10.15. The hemoglobin was 12.4 g/dL, calcium 9.0 mg/dL, serum albumin 3.5 g/dL, and lipide profile was normal. Serum levels of immunoglobulin A (IgA), IgG, and IgM were decreased (596, 68, and 21 mg/dL, respectively). His C3 level was low at 74 mg/dL (normal range: 90-180 mg7dL), and his C4 level was normal (15 mg/dL). An expanded panel of complement testing was not performed. No other changes in the immune study were detected (negative antibodies anti-GBM, ANAs, anti-dsDNA, ANCAs, and cryoglobulins) and the viral serologies for hepatitis B, hepatitis C, and HIV were negative. Respiratory virus (SARS-CoV2, influenza A/B, and parainfluenza) and blood cultures were also negative. There was no evidence of neoplastic disease in the thorax, abdomen, and pelvis on CT-scan. The bone marrow aspirate showed 3.6% of plasma cells, 95% of these with an abnormal phenotype.

**Figure 1:**
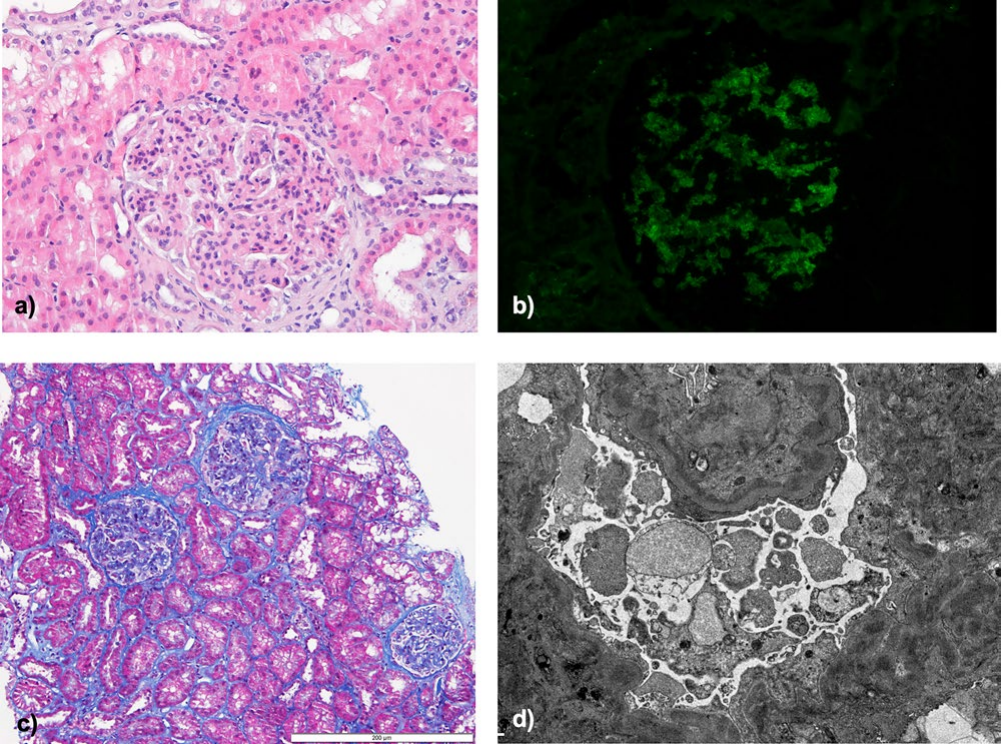
Kidney biopsy. A and C) Optical microscopy (HE and Masson’s trichrome staining, respectively) showing a membranoproliferative pattern, with endocapillary hypercellularity, lobulation, and thickened glomerular basement membranes; B) Immunofluorescence with C3 staining; D) Electron microscopy – mesangial, mesangiocapillary, and subendothelial immune-type deposits.

Renal biopsy showed mesangiocapillary proliferation with a membranoproliferative pattern on light microscopy, with endocapillary hypercellularity, lobulation, and thickened glomerular basement membranes with double contours and tubular atrophy and interstitial fibrosis of 5 and 10%, respectively. Immunofluorescence (IF) revealed mesangial and capillary wall positivity for C3 (2+). C1q was vestigial, with negative staining for immunoglobulins (IgG, IgA, IgM) or light chains κ and λ. In electron microscopy, mesangial, mesangiocapillary, and subendothelial immune-type deposits were observed (Figure 1).

A diagnosis of C3GN associated with monoclonal gammopathy of renal significance (MGRS) was made, and the patient was started on clone-directed therapy in June 2022. He received 7 months of therapy with iv cyclophosphamide 300 mg/m^2^, bortezomib (1.3 mg/m2 weekly), and dexamethasone (40 mg weekly).

Two months after starting chemotherapy, a decrease in the M protein to 0.2 mg/dL and a reduction of free light chain (FLC) ratio kappa/ lambda to 2.2, along with an improvement of proteinuria to 1.6 g/ 24 hours were detected. At the end of the treatment, he achieved a complete hematologic response, with disappearance of the monoclonal protein on serum electrophoresis, normalization of the serum kappa/ lambda ratio, and no detection of monoclonal components on urinary electrophoresis/ immunofixation. Proteinuria further improved to 0.33 g/24 hours, erythrocyturia disappeared, and creatinine remained stable at 1.0 mg/dL.

**Figure 2:**
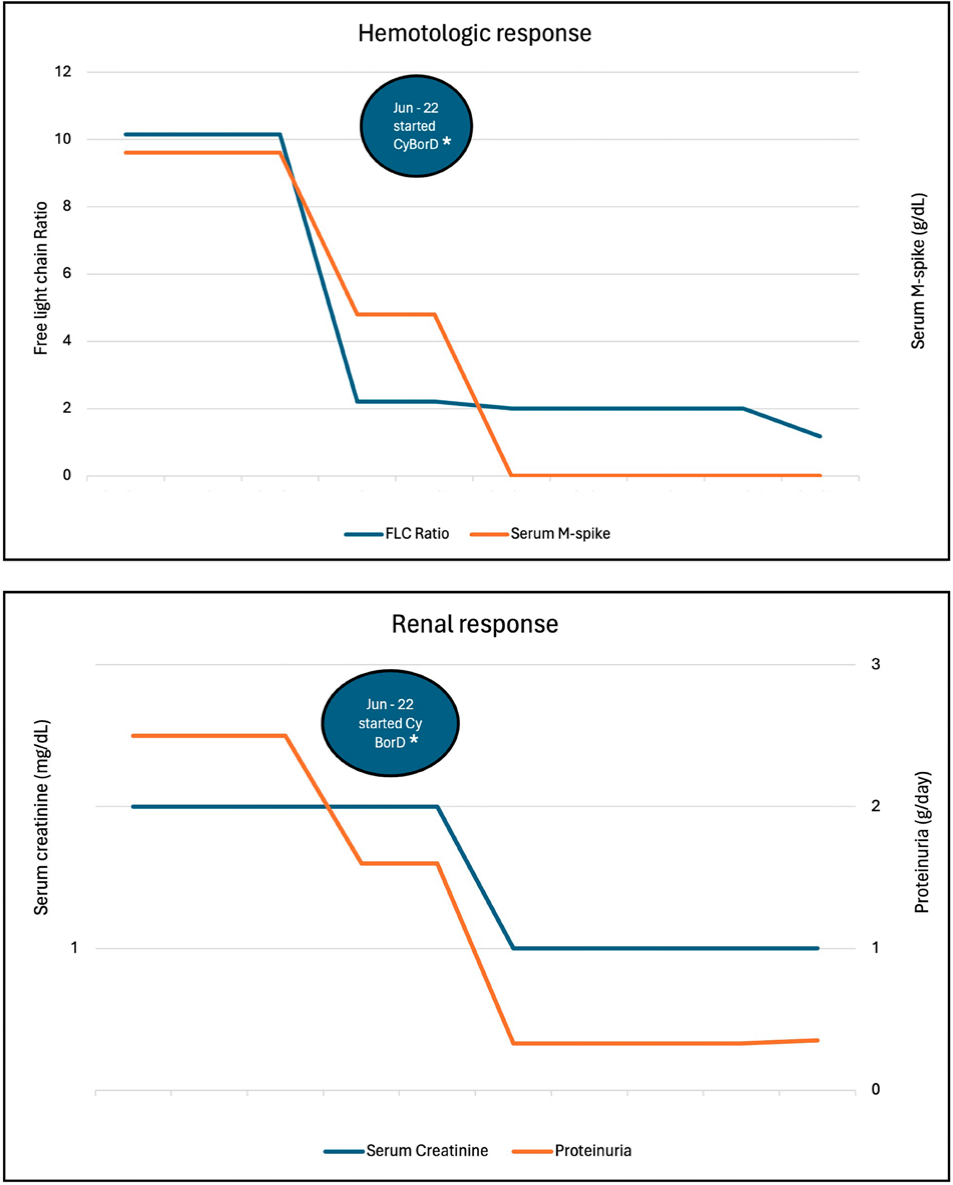
Hematologic and renal responses.

He received no maintenance therapy and remains stable with complete hematologic response, preserved renal function, and proteinuria below 400 mg/day 12 months after stopping the treatment (Figure 2).

*Cyclophosphamide, bortezomib, and dexamethasone.

## Discussion

MGRS is a complex and challenging condition characterized by kidney damage induced by the secretion of a nephrotoxic monoclonal immunoglobulin (mIg). The pattern of renal lesion is mostly determined by the intrinsic structural and physicochemical characteristics of the monoclonal protein (intact monoclonal immunoglobulins or immunoglobulin light chains), rather than by the rate of production or clone features^1,2^. This case report highlights the association between MGRS and C3GN.

C3 glomerulopathies are a group of rare kidney diseases driven by the dysregulation of the complement alternative pathway^7^. C3G is characterized histopathologically by the accumulation of the C3 component in renal tissue. This finding, in the absence or near absence of immunoglobulin deposits, is the single diagnostic criterion. Terminal pathway dysregulation might also occur, especially in C3GN^7^.

Dysregulation of the AP may result from acquired or genetic changes. In most patients, the disease is caused by acquired factors, namely autoantibodies that target regulatory proteins of the complement system. The most common are C3 nephritic factors, which stabilize C3 convertase, increasing its half-life, but other autoantibodies have been identified such as C5 nephritic factors (which target C3bBbC3b), C4 nephritic factors (towards C4b2a), factor H and factor B autoantibodies. Genetic causes are less frequent and include mutations that result either in loss of function in genes responsible for regulatory proteins or in gain of function in activator proteins^7,10,11^.

More recently, an association between monoclonal gammopathy and C3G has been described^4,7^. The pathophysiology of mIg-C3G is still being investigated, but evidence suggests an association between mIg and inappropriate activation of the AP pathway: monoclonal light chains can act as autoantibodies against factor H, resulting in decreased factor H activity, and mIg itself could act as C3 nephritic factor^7,10^. However, the monoclonal immunoglobulin does not always show antibody activity against complement regulatory proteins and is thought to activate the alternative pathway through other mechanisms^4,10,12^. Genetic abnormalities in complement genes are rarely identified in mIg-C3G^4,7,12^.

Clinical presentation of C3G ranges from asymptomatic hematuria and proteinuria of variable degrees, including the nephrotic range, to an acute presentation with nephritic syndrome, acute kidney injury, or rapidly progressive glomerulonephritis^7^. Serum C3 levels are low in most patients, while C4 levels are usually normal; elevated serum levels of sC5b-9 may be present^4,7^. Patients with mIg-C3G are typically older compared to C3G without monoclonal Ig and tend to have higher serum creatinine and proteinuria at presentation^4^.

Renal biopsy is mandatory to establish the diagnosis^7^. All patients with MGUS with suspected kidney involvement, i.e. suspected MGRS (for example, presenting with proteinuria, hematuria, and/ or unexplained kidney function impairment), should undergo a kidney biopsy since the renal diagnosis can dramatically change the therapeutic and prognostic landscape of the disease. For the same reason, kidney biopsy is also recommended in patients with MGUS and other known risk factors for chronic kidney disease who have an atypical clinical course^1^. This case exemplifies the significant impact of a timely diagnosis coupled with effective treatment on the renal prognosis and course of the disease.

On light microscopy, C3G usually shows a membranoproliferative pattern, but can also show mesangial or endocapillary proliferation. Exudative, crescentic, and sclerosing patterns can be observed. The two major subtypes of C3 glomerulopathy, DDD and C3GN, are distinguished by their ultrastructural appearance: ill-defined, moderately electron-dense mesangial, subepithelial and subendothelial deposits are seen in C3GN, whereas highly electron-dense ‘sausage-like’ intramembranous deposits and mesangial rounded nodular deposits are seen in DDD. Subepithelial humps can occur in both subtypes ^7,11^. Immunofluorescence microscopy, key for diagnosis, shows staining for C3 of at least two times greater intensity than for any other immunoreactant and is typically negative for Ig (either heavy or light chains)^7,13^.

C3GN is the most common form of C3 glomerulopathy with monoclonal gammopathy. Importantly, a minority of patients (5–10%) with monoclonal gammopathy and findings on standard immunofluorescence consistent with C3GN will actually have a membranoproliferative glomerulonephritis with masked monoclonal deposits. These patients require additional immunofluorescence studies to be performed on protease-digested, paraffin-embedded tissue for identification of the monoclonal immunoglobulin in the deposits^1^. The limited availability of these auxiliary techniques in our country precluded their utilization in this case.

Moreover, renal biopsy provides valuable prognostic information^7,14^. The Columbia University C3G histologic index, validated in 2017, assesses disease activity and chronicity based on histological features, including mesangial hypercellularity, endocapillary proliferation, membranoproliferative morphology, cellular and fibrocellular crescents, fibrinoid necrosis, and interstitial inflammation (on a scale of 0–3). The features graded for the chronicity score include glomerulosclerosis, tubular atrophy, interstitial fibrosis (each on a scale of 0–3), and arteriosclerosis (on a scale of 0–1). In the study of Bomback et al., the estimated glomerular filtration rate at diagnosis, percent tubular atrophy, and percent interstitial fibrosis were the strongest independent predictors of progression to loss of kidney function^14^. In a more recent, yet smaller, study from Caravaca-Fontán et al., the C3G histologic index was applied to 23 patients with C3G and monoclonal gammopathy, with higher chronicity scores being associated with worse kidney outcomes^12^. Notably, higher chronicity scores at the time of kidney biopsy may indicate a delayed diagnosis in this older population with mIg-C3G^12^. Thus, although challenging, an early diagnosis, before chronic lesions develop, is a crucial prognostic factor, enabling therapeutic interventions to be effective at the renal parenchyma level.

Many older patients, especially those aged ≥50 years, who present with C3G will have a monoclonal gammopathy, indicative of MGRS^4,12,15^. In a large cohort from Mayo Clinic, monoclonal gammopathy was identified in only 8 of 52 (15%) patients <50 years of age, compared with 28 of 43 (65%) patients >50 years of age^15^. Thus, all patients aged ≥50 years with C3G should be screened for paraproteins by serum protein electrophoresis immunofixation and serum FLC evaluation. A diagnosis of monoclonal gammopathy requires further evaluation with bone marrow biopsy to identify the clonal population responsible for mIg production^1,7^. The most common underlying hematologic disease in patients with mIg-G3C is MGUS/ MGRS, and IgG/κ is the most common mIg isotype^2,4^.

Expert opinion suggests that patients with C3G should undergo a comprehensive complement evaluation, including overall complement activity assessment, measurements of serum levels of complement proteins and their split products, and screening for autoantibodies^7^. Genetic testing should also be considered, although its precise value in the clinical setting of patients with mIg-C3G remains to be determined^7,12^. This is a limitation of our study, as neither autoantibody screening nor complement genetic studies were conducted. Nonetheless, the lack of this information doesn’t appear to have significantly affected the diagnostic and prognostic evaluation of our patient, as evidenced by his excellent response to treatment.

The best treatment for C3GN-associated with MGRS has not been established^7^. However, there is increasing evidence of superiority of clone-targeted therapies in terms of kidney survival compared with conventional immunosuppression or conservative management, highlighting the correlation between the reduction of mIg and better renal outcomes^4,9,12^. Chauvet S et al. found that patients who received chemotherapy, including bortezomib, achieved better renal response than those receiving conservative/immunosuppressive therapy^8^. In that study, the achievement of a hematological response was significantly associated with higher kidney survival^8^. These results were later reproduced by some observational studies, supporting the therapeutic superiority of clone-targeted therapies in terms of kidney survival and the correlation of hematologic response with improvement in proteinuria and renal outcomes^4,9,12,13^. Based on these findings, anti-myeloma agents like bortezomib, lenalidomide, or even daratumumab, should be considered as first-line therapy for C3G-MIg until further data are available. In normal clinical practice, it is sometimes difficult to encourage hematologists to perform this type of treatment in the absence of a neoplastic process or clear evidence of mIg deposition in kidney tissue, which is a challenge in the management of these patients. The potential role of newer complement-targeted therapies as adjunctive treatment in selected patients with mIg-C3G is yet to be assessed^7^.

Similarly, the optimal treatment for C3G without mIg has not been established. Most of these patients are treated with conventional immunosuppression (corticosteroids alone or corticosteroids plus other drugs), with mixed results^7^. In a study by Ravindran et al, there was no significant difference in renal survival among patients with mIg-C3G and patients with C3G without mIg^4^. In the specific setting of mIg-C3G, clone-directed therapy may result in improved renal survival^4,7,9,13^.

The limited data on mIg-C3G in kidney transplant point to a high risk of disease recurrence in allograft recipients^7^. Besides, transplant recipients with C3G-MIg seem to have poor kidney outcomes despite the achievement of hematological response in a few cases^12^. In younger, selected patients who are transplant candidates, autologous stem cell transplant could be considered as a complementary therapy, helping to achieve a deeper and sustained or even a complete hematological response, which is essential to reduce the risk of recurrence, particularly after renal transplant^16^.

The treatment approach adopted in this case was clone-directed therapy. Remarkably, the patient achieved complete hematologic response with the disappearance of M-spike on serum electrophoresis and normalization of the serum FLC kappa/ lambda ratio. The success of clone-directed therapy in this case highlights the importance of tailored treatment strategies in MGRS-associated C3G, in agreement with the most recent evidence. Furthermore, diagnosis was established early, with few signs of chronicity on renal biopsy. This, coupled with the efficacy of the clone-direct therapy, markedly influenced the patient’s renal prognosis. The long-term follow-up revealed sustained remission of the hematologic condition and proteinuria, while maintaining preserved renal function, reinforcing that achieving a complete or deep hematologic response results in improved kidney outcomes.

## Conclusions

This case underscores the intricate relationship between MGRS and C3GN, highlighting the importance of tailored treatment strategies. The patient exhibited an exceptional response to treatment, demonstrating the importance of early diagnosis and effective clone-directed therapy in improving renal prognosis. The long-term remission of both hematologic and renal parameters emphasizes the importance of clone-directed therapy and the need for vigilant follow-up in managing MGRS-associated C3G cases.
