# Supplementary material for: C3 glomerulonephritis associated with monoclonal gammopathy of renal significance: a diagnostic and therapeutic challenge
Source: J Bras Nefrol. 2025 Feb 7;47(2):e20240106. doi: 10.1590/2175-8239-JBN-2024-0106en (PMC11804883; doi:10.1590/2175-8239-JBN-2024-0106en)
Supplement: Translation

# Translation: Glomerulonefrite C3 associada à gamopatia monoclonal de significância renal: um desafio diagnóstico e terapêutico

**DOI:** 10.1590/2175-8239-JBN-2024-0106pt

**Authors:** Bárbara Beirão, Mariana Freitas, Natália Silva, Patrícia Ferraz, Catarina Prata, Teresa Morgado

**Affiliations:** 1 Centro Hospitalar de Trás-os-Montes e Alto Douro, Hospital de Vila Real, Serviço de Nefrologia, Vila Real, Portugal.; 2 Centro Hospitalar de Trás-os-Montes e Alto Douro, Hospital de Vila Real, Serviço de Hematologia, Vila Real, Portugal.

**Keywords:** Gamopatia Monoclonal, Glomerulopatia C3, Gamopatia Monoclonal de Significado Renal

## Abstract

As glomerulopatias C3 constituem um grupo heterogêneo de glomerulopatias caracterizadas por desregulação da via alternativa do complemento. Embora a fisiopatologia não esteja completamente esclarecida, há um reconhecimento crescente da associação entre essa pato­logia e gamopatias monoclonais, especial­mente em indivíduos mais velhos. Ainda há alguma incerteza em relação ao melhor tratamento para doentes com glomerulopatia C3 associada a gamopatia monoclonal. No entanto, evidência recente sugere que tratamentos dirigidos ao clone monoclonal estão associados a melhores desfechos renais em comparação com terapias conservadoras ou imunossupressão convencional. Este relato de caso descreve o curso clínico e seguimento de longo prazo de um doente com glomerulonefrite C3 associada a gamopatia monoclonal tratado com terapêutica antimieloma.

## Introdução

O termo gamopatia monoclonal de significância renal (GMSR) descreve condições hematológicas em que a produção e secreção de uma imunoglobulina monoclonal (IgM) causa danos renais^1^. Esses distúrbios hematológicos não preenchem os critérios diagnósticos de mieloma múltiplo ou doença linfoproliferativa e, portanto, não preenchem os critérios hematológicos previamente definidos para tratamento direcionado^1^.

O espectro das doenças renais associadas à GMSR é amplo. A injúria renal pode ser causada diretamente pela deposição da proteína monoclonal ou, menos comumente, pela desregulação da via alternativa (VA) do complemento^1–3^. Nesse mecanismo indireto, a Ig monoclonal atua como um autoanticorpo, ativando a VA por meio da inibição de proteínas reguladoras do complemento, sem que haja depósitos de Ig nos rins^1,3^. O melhor exemplo de um distúrbio associado à GMSR com deposição ausente ou escassa de Ig monoclonal é a glomerulopatia C3 (GC3), incluindo a doença de depósito denso (DDD) e a glomerulonefrite C3 (GNC3)^1^.

Embora a GC3 seja rara, vários estudos demonstraram uma alta prevalência de gamopatia monoclonal em pacientes com mais de 50 anos com GC3 (chegando a 65% em algumas séries)^4^, muito superior à prevalência de gamopatia monoclonal na população em geral (4,2%)^5,6^.

A GC3 é um diagnóstico histopatológico, caracterizado pela deposição dominante de C3 na coloração por imunofluorescência. A apresentação é variável, incluindo desde hematúria assintomática até glomerulonefrite rapidamente progressiva^7^.

Atualmente, ainda existe alguma incerteza quanto ao melhor tratamento para pacientes com GNC3 associada à IgM (mIg-C3GN, por sua sigla em inglês). No entanto, as evidências de algumas séries e relatos de casos sugerem que as terapias direcionadas ao mieloma estão associadas a melhores desfechos renais em comparação com as terapias imunossupressoras conservadoras ou convencionais^4,8,9^.

Este relato de caso descreve o curso clínico e os desfechos de um paciente com GMSR associada à GNC3 tratado com terapia direcionada ao clone.

## Relato de Caso

Um homem de 61 anos se apresentou ao nosso serviço com histórico de insuficiência cardíaca (FEVE 46%) após duas internações por miopericardite com choque cardiogênico em 2018 e 2021. A biópsia miocárdica realizada em 2018 revelou um infiltrado linfocitário discreto e inespecífico. Não havia evidência de etiologia imunológica ou neoplásica e presumiu-se um diagnóstico de miopericardite viral. Ele apresentava gamopatia monoclonal de significância indeterminada (GMSI) do tipo IgG/kappa (pico M de 0,38 g/dL na eletroforese/imunofixação de proteínas séricas e razão de cadeias leves livres kappa/ lambda de 5,47).

Em janeiro de 2022, ele apresentou eritrocitúria de 25–50/hpf, proteinúria de 2,5 g/dia (albuminúria 1700 mg/dia) e função renal preservada (CrS 1,1/ Ur 65 mg/dL, depuração de creatinina 94 mL/min). O diagnóstico de gamopatia monoclonal IgG/kappa foi estabelecido com base nos resultados da imunofixação de soro e urina: proteína M de 0,4 g/dL, nível sérico da cadeia leve kappa de 11,8 mg/L e nível sérico da cadeia leve lambda de 1,16 mg/L, com uma razão correspondente de 10,15. A hemoglobina era de 12,4 g/dL, cálcio de 9,0 mg/dL, albumina sérica de 3,5 g/dL e o perfil lipídico estava dentro da normalidade. Os níveis séricos de imunoglobulina A (IgA), IgG e IgM estavam reduzidos (596, 68 e 21 mg/dL, respectivamente). Seu nível de C3 estava baixo, em 74 mg/dL (intervalo normal: 90–180 mg/dL) e seu nível de C4 estava normal (15 mg/dL). Não foi realizado um painel expandido para testes de complemento. Não foram detectadas outras alterações no estudo imunológico (anticorpos anti-GBM, ANAs, anti-dsDNA, ANCAs e crioglobulinas negativos) e as sorologias virais para hepatite B, hepatite C e HIV foram negativas. Os vírus respiratórios (SARS-CoV2, influenza A/B e parainfluenza) e as hemoculturas também foram negativos. Não houve evidência de doença neoplásica no tórax, abdome e pelve na tomografia computadorizada. O aspirado de medula óssea revelou 3,6% de células plasmáticas, das quais 95% apresentavam um fenótipo anormal.

A biópsia renal mostrou proliferação mesangiocapilar com um padrão membranoproliferativo à microscopia óptica, com hipercelularidade endocapilar, lobulação e membranas basais glomerulares espessadas com contornos duplos, e atrofia tubular e fibrose intersticial de 5 e 10%, respectivamente. A imunofluorescência (IF) revelou positividade mesangial e da parede capilar para C3 (2+). C1q era vestigial, com coloração negativa para imunoglobulinas (IgG, IgA, IgM) ou cadeias leves κ e λ. Na microscopia eletrônica, foram observados depósitos do tipo imune mesangiais, mesangiocapilares e subendoteliais (Figura 1).

**Figura 1:** Biópsia renal. A e C) Microscopia óptica (coloração HE e tricrômico de Masson, respectivamente) mostrando padrão membranoproliferativo, com hipercelularidade endocapilar, lobulação e membranas basais glomerulares espessadas; B) Imunofluorescência com coloração para C3; D) Microscopia eletrônica - depósitos mesangiais, mesangiocapilares e subendoteliais do tipo imune.

Foi feito um diagnóstico de GNC3 associada à gamopatia monoclonal de significância renal (GMSR), e o paciente iniciou a terapia direcionada ao clone em junho de 2022. Ele recebeu 7 meses de terapia com ciclofosfamida iv 300 mg/m^2^, bortezomibe (1,3 mg/m^2^ semanalmente) e dexametasona (40 mg semanalmente).

Dois meses após o início da quimioterapia, detectou-se uma redução da proteína M para 0,2 mg/dL e uma diminuição da razão de cadeias leves livres (CLL) kappa/ lambda para 2,2, juntamente com uma melhora da proteinúria para 1,6 g/24 horas. Ao final do tratamento, o paciente obteve uma resposta hematológica completa, com desaparecimento da proteína monoclonal na eletroforese sérica, normalização da razão kappa/ lambda sérica e nenhuma detecção de componentes monoclonais na eletroforese/imunofixação urinária. A proteinúria melhorou ainda mais, para 0,33 g/24 horas, a eritrocitúria desapareceu e a creatinina permaneceu estável em 1,0 mg/dL.

Ele não recebeu terapia de manutenção e permanece estável com resposta hematológica completa, função renal preservada e proteinúria abaixo de 400 mg/dia 12 meses após a interrupção do tratamento (Figura 2).

**Figura 2:** Respostas hematológicas e renais.

*Ciclofosfamida, bortezomibe e dexametasona.

## Discussão

A GMSR é uma condição complexa e desafiadora caracterizada por lesão renal induzida pela secreção de uma imunoglobulina monoclonal (IgM) nefrotóxica. O padrão de lesão renal é determinado principalmente pelas características estruturais e fisioquímicas intrínsecas da proteína monoclonal (imunoglobulinas monoclonais intactas ou cadeias leves de imunoglobulina), e não pela taxa de produção ou características do clone^1,2^. Este relato de caso destaca a associação entre GMSR e GNC3.

As glomerulopatias C3 são um grupo de doenças renais raras causadas pela desregulação da via alternativa do complemento^7^. A GC3 é caracterizada histopatologicamente pelo acúmulo do componente C3 no tecido renal. Esse achado, na ausência ou quase ausência de depósitos de imunoglobulina, é o único critério diagnóstico. A desregulação da via terminal também pode ocorrer, especialmente na GNC3^7^.

A desregulação da VA pode resultar de alterações adquiridas ou genéticas. Na maioria dos pacientes, a doença é causada por fatores adquiridos, ou seja, autoanticorpos que têm como alvo proteínas reguladoras do sistema complemento. Os mais comuns são os fatores nefríticos do C3, que estabilizam a C3 convertase, aumentando sua meia-vida. No entanto, outros autoanticorpos foram identificados, como os fatores nefríticos do C5 (que têm como alvo o C3bBbC3b), os fatores nefríticos do C4 (direcionados ao C4b2a), além de autoanticorpos contra o fator H e o fator B. As causas genéticas são menos frequentes e incluem mutações que resultam em perda de função nos genes responsáveis por proteínas reguladoras ou em ganho de função nas proteínas ativadoras^7,10,11^.

Mais recentemente, foi descrita uma associação entre gamopatia monoclonal e GC3^4,7^. A fisiopatologia da mIg-C3G ainda está sob investigação, mas as evidências sugerem uma associação entre a IgM e a ativação inadequada da VA: as cadeias leves monoclonais podem atuar como autoanticorpos contra o fator H, resultando em redução da atividade do fator H, e a própria IgM pode atuar como fator nefrítico do C3^7,10^. Entretanto, a imunoglobulina monoclonal nem sempre apresenta atividade de anticorpo contra proteínas reguladoras do complemento e acredita-se que ative a via alternativa por meio de outros mecanismos^4,10,12^. Anormalidades genéticas em genes do complemento raramente são identificadas na mIg-C3G^4,7,12^.

A apresentação clínica da GC3 varia desde hematúria assintomática e proteinúria de graus variáveis, incluindo a faixa nefrótica, até uma apresentação aguda com síndrome nefrítica, injúria renal aguda ou glomerulonefrite rapidamente progressiva^7^. Os níveis séricos de C3 são baixos na maioria dos pacientes, enquanto os níveis de C4 são geralmente normais; níveis séricos elevados de sC5b-9 podem estar presentes^4,7^. Os pacientes com mIg-C3G são geralmente mais velhos em comparação com GC3 sem Ig monoclonal e tendem a exibir níveis mais elevados de creatinina sérica e proteinúria na apresentação^4^.

A biópsia renal é obrigatória para estabelecer o diagnóstico^7^. Todos os pacientes com GMSI com suspeita de envolvimento renal, ou seja, suspeita de GMSR (por exemplo, apresentando proteinúria, hematúria e/ou comprometimento inexplicável da função renal), devem ser submetidos a uma biópsia renal, uma vez que o diagnóstico renal pode alterar drasticamente o panorama terapêutico e prognóstico da doença. Pelo mesmo motivo, a biópsia renal também é recomendada em pacientes com GMSI e outros fatores de risco conhecidos para doença renal crônica que apresentem um curso clínico atípico^1^. Este caso exemplifica o impacto significativo de um diagnóstico oportuno, associado a um tratamento eficaz, no prognóstico renal e no curso da doença.

Na microscopia óptica, a GC3 geralmente apresenta um padrão membranoproliferativo, mas também pode apresentar proliferação mesangial ou endocapilar. Podem ser observados padrões exsudativos, crescênticos e esclerosantes. Os dois principais subtipos de glomerulopatia C3, DDD e GNC3, são diferenciados por sua aparência ultraestrutural: depósitos mesangiais, subepiteliais e subendoteliais difusos e moderadamente densos em elétrons são observados na GNC3, enquanto depósitos intramembranosos altamente densos em elétrons, em forma de “salsicha” e depósitos mesangiais nodulares arredondados são observados na DDD. Podem ocorrer depósitos subepiteliais, os *“humps”*, em ambos os subtipos^7,11^. A microscopia de imunofluorescência, fundamental para o diagnóstico, mostra coloração para C3 com intensidade pelo menos duas vezes maior do que para qualquer outro imunorreagente e é tipicamente negativa para Ig (cadeias pesadas ou leves)^7,13^.

A GNC3 é a forma mais comum de glomerulopatia C3 com gamopatia monoclonal. É importante ressaltar que uma minoria de pacientes (5 a 10%) com gamopatia monoclonal e achados na imunofluorescência padrão consistentes com GNC3 terão, de fato, uma glomerulonefrite membranoproliferativa com depósitos monoclonais mascarados. Esses pacientes precisam de estudos adicionais de imunofluorescência a serem realizados em tecido digerido por protease e embebido em parafina para a identificação da imunoglobulina monoclonal nos depósitos^1^. A disponibilidade limitada dessas técnicas auxiliares em nosso país inviabilizou sua utilização neste caso.

Além disso, a biópsia renal fornece informações prognósticas valiosas^7,14^. O índice histológico de GC3 da *Columbia University*, validado em 2017, avalia a atividade e cronicidade da doença com base em características histológicas, incluindo hipercelularidade mesangial, proliferação endocapilar, morfologia membranoproliferativa, crescentes celulares e fibrocelulares, necrose fibrinoide e inflamação intersticial (em uma escala de 0–3). As características avaliadas para o escore de cronicidade incluem glomerulosclerose, atrofia tubular, fibrose intersticial (cada uma em uma escala de 0–3) e arteriosclerose (em uma escala de 0-1). No estudo de Bomback et al., a taxa de filtração glomerular estimada no momento do diagnóstico, o percentual de atrofia tubular e o percentual de fibrose intersticial foram os preditores independentes mais fortes da progressão para a perda da função renal^14^. Em um estudo mais recente, embora menor, de Caravaca-Fontán et al., o índice histológico de GC3 foi aplicado a 23 pacientes com GC3 e gamopatia monoclonal, com escores de cronicidade mais elevados associados a piores desfechos renais^12^. Notavelmente, escores de cronicidade mais elevados no momento da biópsia renal podem indicar um diagnóstico tardio nessa população com idade mais avançada com mIg-C3G^12^. Portanto, embora desafiador, um diagnóstico precoce, anterior ao desenvolvimento de lesões crônicas, é um fator prognóstico crucial, permitindo que as intervenções terapêuticas sejam eficazes no nível do parênquima renal.

Muitos pacientes mais velhos, especialmente aqueles com idade ≥50 anos que apresentam GC3, terão gamopatia monoclonal, indicativa de GMSR^4,12,15^. Em uma grande coorte da *Mayo Clinic*, a gamopatia monoclonal foi identificada em apenas 8 de 52 (15%) pacientes < 50 anos de idade, em comparação com 28 de 43 (65%) pacientes > 50 anos de idade^15^. Portanto, todos os pacientes com idade ≥ 50 anos com GC3 devem ser triados para paraproteínas por eletroforese de proteínas séricas, imunofixação e avaliação de CLL séricas. O diagnóstico de gamopatia monoclonal requer avaliação adicional com biópsia da medula óssea para identificar a população clonal responsável pela produção de IgM^1,7^. A doença hematológica subjacente mais comum em pacientes com mIg-G3C é a GMSI/GMSR, sendo IgG/κ o isótipo mais comum de IgM^2,4^.

A opinião de especialistas sugere que os pacientes com GC3 devem ser submetidos a uma avaliação abrangente do complemento, incluindo avaliação geral da atividade do complemento, medições dos níveis séricos das proteínas do complemento e de seus produtos de clivagem e triagem de autoanticorpos^7^. Testes genéticos também devem ser considerados, embora seu valor preciso no cenário clínico de pacientes com mIg-C3G ainda não tenha sido determinado^7,12^. Essa é uma limitação do nosso estudo, uma vez que não foram realizados nem a triagem para autoanticorpos nem estudos genéticos do complemento. No entanto, a falta dessas informações não parece ter afetado significativamente a avaliação diagnóstica e prognóstica de nosso paciente, conforme evidenciado por sua excelente resposta ao tratamento.

O melhor tratamento para a GNC3 associada à GMSR ainda não foi estabelecido^7^. No entanto, há evidências crescentes da superioridade das terapias direcionadas ao clone em termos de sobrevida renal em comparação com a imunossupressão convencional ou o manejo conservador, destacando a correlação entre a redução de IgM e melhores desfechos renais^4,9,12^. Chauvet S et al. constataram que os pacientes que receberam quimioterapia, incluindo bortezomibe, obtiveram melhor resposta renal do que aqueles que receberam terapia conservadora/imunossupressora^8^. Nesse estudo, a obtenção de uma resposta hematológica foi significativamente associada a uma maior sobrevida renal^8^. Esses resultados foram posteriormente reproduzidos por alguns estudos observacionais, corroborando a superioridade terapêutica das terapias direcionadas ao clone em termos de sobrevida renal e a correlação da resposta hematológica com a melhoria da proteinúria e dos desfechos renais^4^. Com base nesses achados, agentes antimieloma, como bortezomibe, lenalidomida ou até mesmo daratumumabe, devem ser considerados como terapia de primeira linha para mIg-C3G até que mais dados estejam disponíveis. Na prática clínica normal, é difícil, às vezes, incentivar os hematologistas a realizarem esse tipo de tratamento na ausência de um processo neoplásico ou de evidências claras de deposição de IgM no tecido renal, o que representa um desafio no tratamento desses pacientes.

O papel potencial das mais novas terapias direcionadas ao complemento como tratamento adjuvante em pacientes selecionados com mIg-C3G ainda não foi avaliado^7^.

Da mesma forma, o tratamento ideal para a GC3 sem IgM não foi estabelecido. A maioria desses pacientes é tratada com imunossupressão convencional (corticosteroides isolados ou corticosteroides associados a outros medicamentos), com resultados variados^7^. Em um estudo realizado por Ravindran et al., não houve diferença significativa na sobrevida renal entre pacientes com mIg-C3G e pacientes com GC3 sem IgM^4^. No contexto específico da mIg-C3G, a terapia direcionada ao clone pode resultar em melhora da sobrevida renal^4^.

Os dados limitados sobre mIg-C3G no transplante renal apontam para um risco elevado de recidiva da doença em receptores de aloenxertos^7^. Além disso, os receptores de transplante com C3G-mIg parecem apresentar piores desfechos renais, apesar da obtenção de resposta hematológica em alguns casos^12^. Em pacientes mais jovens e selecionados, que são candidatos a transplante, o transplante autólogo de células-tronco pode ser considerado uma terapia complementar, ajudando a alcançar uma resposta hematológica mais profunda e sustentada ou até mesmo completa, o que é essencial para reduzir o risco de recidiva, principalmente após o transplante renal^16^.

A abordagem terapêutica adotada neste caso foi a terapia direcionada ao clone. Notavelmente, o paciente obteve resposta hematológica completa com o desaparecimento do pico M na eletroforese de proteínas séricas e normalização da razão de CLL kappa/ lambda no soro. O sucesso da terapia direcionada ao clone neste caso destaca a importância de estratégias de tratamento personalizadas na GC3 associada à GMSR, em conformidade com as evidências mais recentes (ref). Além disso, o diagnóstico foi estabelecido precocemente, com poucos sinais de cronicidade na biópsia renal. Isso, aliado à eficácia da terapia direcionada ao clone, influenciou de maneira significativa o prognóstico renal do paciente. O acompanhamento de longo prazo revelou remissão sustentada da condição hematológica e da proteinúria, além de manter a função renal preservada, reforçando que a obtenção de uma resposta hematológica completa ou profunda resulta em melhores desfechos renais.

## Conclusões

Este caso ressalta a complexa relação entre GMSR e GNC3, destacando a importância de estratégias de tratamento personalizadas. O paciente apresentou uma resposta excepcional ao tratamento, demonstrando a importância do diagnóstico precoce e da terapia eficaz direcionada ao clone na melhora do prognóstico renal. A remissão a longo prazo dos parâmetros hematológicos e renais enfatiza a importância da terapia direcionada ao clone e a necessidade de acompanhamento vigilante no manejo dos casos de GC3 associados à GMSR.
